# A Hierarchical Dispatcher for Scheduling Multiple Deep Neural Networks (DNNs) on Edge Devices

**DOI:** 10.3390/s25072243

**Published:** 2025-04-02

**Authors:** Hyung Kook Jun, Taeho Kim, Sang Cheol Kim, Young Ik Eom

**Affiliations:** 1 Electronics and Telecommunications Research Institute, 218 Gajeong-ro, Yuseong-gu, Daejeon 34129, Republic of Korea; hkjun@etri.re.kr (H.K.J.); sheart@etri.re.kr (S.C.K.); 2 Department of Electrical and Computer Engineering, Sungkyunkwan University, 2066 Seobu-ro, Jangan-gu, Gyeonggi, Suwon 16419, Republic of Korea; yieom@skku.edu; 3 Information Security Division, Seoul Women’s University, 621 Hwarang-ro, Nowon-gu, Seoul 01797, Republic of Korea

**Keywords:** dispatcher, scheduler, deep learning, DNN, edge device

## Abstract

This paper presents a hierarchical dispatcher architecture designed to efficiently schedule the execution of multiple deep neural networks (DNNs) on edge devices with heterogeneous processing units (PUs). The proposed architecture is applicable to systems where PUs are either integrated on a single edge device or distributed across multiple devices. We separate the dispatcher and scheduling policy. The dispatcher in our framework acts as a mechanism for allocating, executing, and managing subgraphs of DNNs across various PUs, and the scheduling policy generates optimized scheduling sequences. We formalize a hierarchical structure consisting of high-level and low-level dispatchers, which together provide scalable and flexible scheduling support for diverse DNN workloads. The high-level dispatcher oversees the partitioning and distribution of subgraphs, while the low-level dispatcher handles the execution and coordination of subgraphs on allocated PUs. This separation of responsibilities allows the architecture to efficiently manage workloads in both homogeneous and heterogeneous environments. Through case studies on edge devices, we demonstrate the practicality of the proposed architecture. By integrating appropriate scheduling policies, our approach achieves an average performance improvement of 51.6%, providing a scalable and adaptable solution for deploying deep learning models on heterogeneous edge systems.

## 1. Introduction

The rapid increase in deep learning models such as deep neural networks (DNNs) has led to extensive research on optimizing their execution while ensuring efficient utilization of computing resources. This includes strategies for allocating, deploying and executing models on edge devices, such as intelligent sensors.

**Figure 1:**
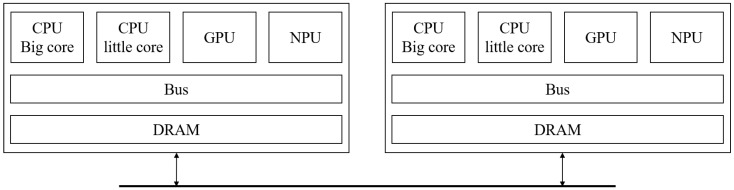
An abstract block diagram of a heterogeneous architecture.

Traditionally, only a single deep learning model was executed on a standalone edge device. However, with the expansion of the scope and the increasing complexity of the deep learning model, the demand to run multiple deep learning models on edge devices has grown significantly. Consequently, it has become essential to efficiently process multiple deep learning models on edge devices equipped with heterogeneous processing units (PUs), including central processing units (CPUs), graphics processing units (GPUs) and neural processing units (NPUs). These PUs can be integrated on a single chip or distributed on multiple chips, as illustrated in Figure 1, requiring advanced resource management and scheduling strategies for optimal execution.

The development of optimized schedulers for general applications on edge devices with heterogeneous processing units (PUs) enables significant improvements in performance, latency, energy efficiency, and real-time requirements [1,2,3].

Similarly, optimized schedulers for deep learning applications can enhance performance, energy efficiency, and so on. A deep learning scheduling strategy partitions deep learning models into submodels and executes them efficiently across multiple PUs. Various scheduling methods have been proposed, each tailored to specific applications and constraints. They are summarized in [4].

In a general-purpose operating system (GPOS) such as Linux and Windows, a scheduler is typically composed of two components: a dispatcher and a scheduling policy. The dispatcher is responsible for managing process switching, ensuring that processes are transferred smoothly between different states of execution. In contrast, the scheduling policy determines the order of process execution by optimizing scheduling sequences to achieve specific objectives such as throughput, latency, fairness, or resource utilization.

Scheduling policies for deep learning have been extensively researched, for example, distributed deep learning partitions data and models into multiple processing units (mostly GPUs) for parallel execution to leverage the computational capacity of many computing nodes in a cluster. Data parallelism [5], model parallelism [6] and pipeline parallelism [7,8,9] have been proposed for optimization techniques. Research has also explored deep learning scheduling strategies aimed at reducing power consumption using DVFS (Dynamic Voltage and Frequency Scaling) on ARM-based Heterogeneous Multi-Processor Systems-on-Chips (HMPSoCs) [10]. The demand for scheduling deep learning models on edge devices has grown rapidly, driven by the increasing complexity of workloads involving multiple PUs and diverse deep learning models operating simultaneously on a single device [11,12].

While extensive research has been conducted on deep learning scheduling strategies, the architectural structure of dispatchers for heterogeneous PUs remains largely underexplored. As a result, most scheduler implementations primarily focus on policy decisions without addressing the underlying dispatcher architecture. This leads to inefficiencies, as dispatchers often require reimplementation whenever a new scheduling policy is introduced. Moreover, existing dispatchers are typically designed for specific hardware platforms, lacking a generalized dispatcher architecture that ensures the flexibility and reusability of DNNs across heterogeneous computing environments. Therefore, there is a clear need for a unified dispatcher architecture capable of supporting a variety of scheduling policies for deep learning workloads on diverse hardware platforms.

In this paper, we present the design and implementation of a dispatcher capable of supporting the scheduling of multiple deep learning models from the perspective of edge devices equipped with multiple PUs. The proposed dispatcher architecture is designed to enhance the capabilities of deep-learning-enabled operating systems, offering scalability and flexibility to efficiently manage complex and heterogeneous workloads.

This paper is structured as follows. Section 2 introduces the dispatcher architecture, and Section 3 presents application case studies to demonstrate its effectiveness. Finally, Section 4 provides the discussion and conclusions.

## 2. A Hierarchical Dispatcher for DNNs

A DNN (deep neural network) scheduler decides how to allocate DNNs to computing resources. The scheduler optimizes the execution of DNN workloads by efficiently allocating various computing resources such as the CPU (central processing unit), GPU (graphical processing unit), and NPU (neural processing unit).

Recently, the size of deep neural network (DNN) models has increased significantly, leading to a mismatch between the computational and memory requirements of these models and the resource constraints of edge devices. Edge devices, typically characterized by limited computational power, memory, and energy, often cannot execute large DNN models directly. Furthermore, the demand to execute multiple DNN models simultaneously on a single edge device or multiple edge devices is increasing, and it introduces the need for allocating submodels on computing resources, executing them, and managing them. These issues pose significant challenges for the deployment of deep learning on edge devices, requiring research such as model compression, partitioning, distributed and federated inference, and dynamic scheduling to bridge the gap between growing models’ complexities and edge devices’ limitations.

The challenges posed by the increasing size of deep neural network (DNN) models and the resource limitations of edge devices can be effectively addressed using deep learning partitioners and deep learning schedulers. Deep learning partitioners, sometimes implemented withina compiler or loader, construct submodels or partitions from DNNs as units of scheduling. On the other hand, multiple DNN models are executed through multiple submodels by allocating, executing, and releasing across PUs. A scheduling policy determines the allocation of tasks to PUs to optimize resource utilization, minimize latency, and meet additional requirements such as real-time constraints.

**Figure 2:**
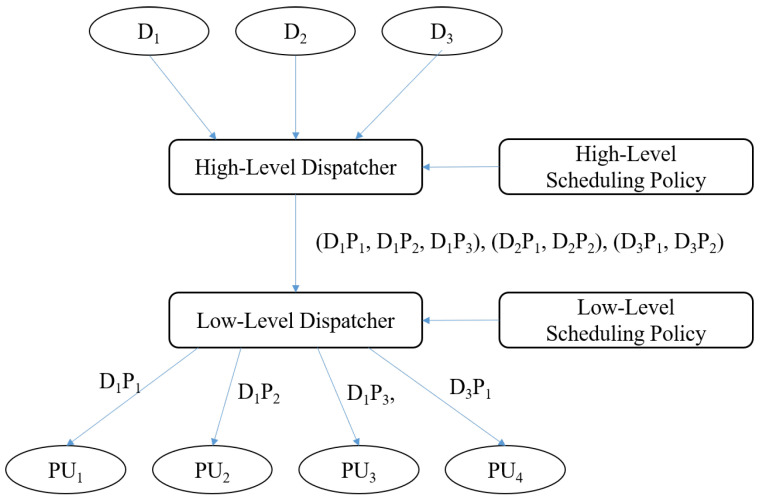
A deep learning dispatcher architecture.

A deep learning dispatcher manages the execution of DNN models by handling two distinctive functionalities: loading DNN models and supporting various scheduling policies. To address the complexities of modern DNN deployments, we propose a two-level deep learning dispatcher architecture to support separated scheduling policies in Figure 2.

**Figure 3:**
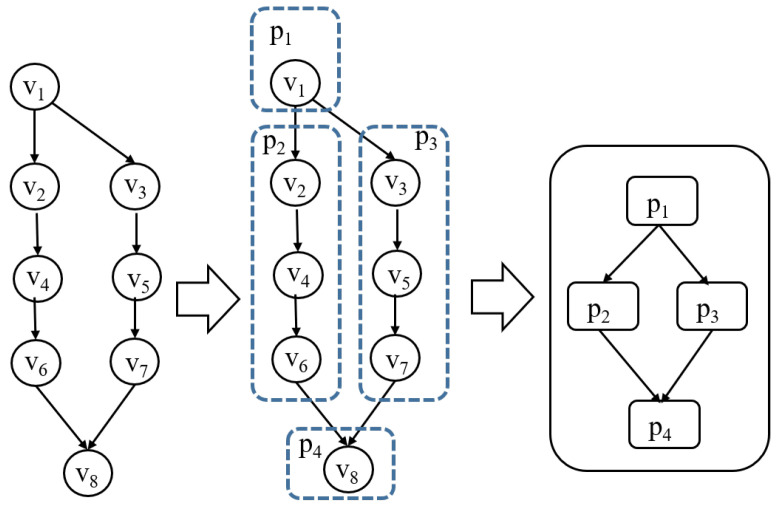
A high-level dispatcher.

**Figure 4:**
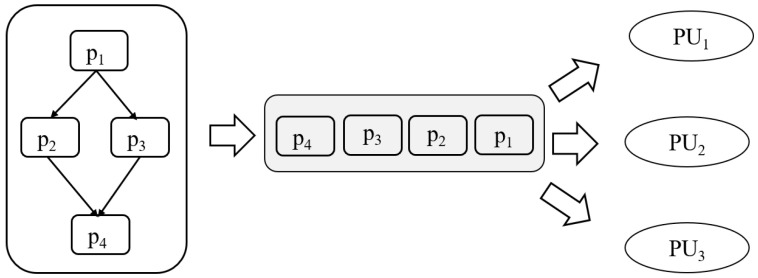
A low-level dispatcher.

**High-Level Dispatcher**  A high-level dispatcher is responsible for constructing a graph of submodels derived from a compiled DNN model. The graph representation captures the dependencies and execution order of submodels, enabling the systematic decomposition of the DNN model into partitions, which act as scheduling units. This abstraction allows for the efficient handling of complex DNN architectures while preparing them for execution. An entering node and an exiting node of a partition are special nodes designated for receiving and sending intermediate (tensor) values, respectively. These nodes not only facilitate data transfer between partitions but also serve as synchronization points. Specifically, the entering node ensures that all required inputs are available before a submodel begins execution, while the exiting node signals the completion of a computation task by transmitting outputs to subsequent partitions. By utilizing these nodes as synchronization nodes, the architecture maintains data consistency and supports asynchronous execution, effectively reducing unnecessary synchronization overhead while ensuring correctness. Figure 3 is an example of a high-level dispatcher. The criteria for partitioning are defined within the high-level scheduling policy, ensuring that partitions are created according to specific performance objectives and resource constraints.
**Low-Level Dispatcher**  Operating at a finer granularity, the low-level dispatcher allocates submodels to processing units (PUs), such as CPUs, GPUs, or NPUs. This allocation is performed in compliance with the selected scheduling policy, ensuring efficient resource utilization and adherence to performance objectives such as latency, throughput, and energy efficiency. Since multiple partitions can be processed simultaneously, PUs function as shared resources and must be released immediately after execution for other partitions to utilize them. Our low-level scheduling operates through the following steps: (1) verifying the maximum PU requirement upon partition input within the system, (2) allocating and executing available PUs, (3) registering the partition in a waiting queue if all PUs are occupied, and (4) releasing PUs upon execution completion and assigning them to waiting partitions. The allocation of partition on different PUs, as depicted in Figure 1, incurs a context switching time during steps (1) and (2). The context switching time includes a communication cost of data through the memory bus or networks, arising from the connection between the sender node and the receiver node. Figure 4 is an example of a low-level dispatcher.

The proposed deep learning dispatcher improves the scalable and flexible execution of DNN models by leveraging this hierarchical structure in heterogeneous edge devices.

### 2.1. Formal Definitions

- The deep learning high-level dispatcher $D_{HL}$ is defined as
  $$D_{HL}:D\to P$$
  where
  1. DNN Graph *D* is
    D=(V,E)
    where
    - The set of vertices representing computational units (e.g., layers, operations, or sub-operations) in the DNN is
      $$V=\{v_{1},v_{2},\ldots ,v_{n}\}$$
    - The set of directed edges representing dependencies between computational units is
      $$E=\{\left(v_{i},v_{j}\right)|v_{i},v_{j}\in V\}$$
  2. The partition of the DNN graph *D* is defined as
    $$P=\{P_{1},P_{2},\ldots ,P_{k}\}$$
    where
    - Each partition $P_{i}$ is a subset of *V*:
      $$P_{i}\subseteq V,\;\forall i\in \left\{1,2,\ldots ,k\right\}$$
    - The union of all partitions covers the entire set of vertices is
      $$\overset{k}{\underset{i=1}{⋃}}P_{i}=V$$
    - Partitions are disjoint:
      $$P_{i}\cap P_{j}=\emptyset ,\;\forall i\neq j$$
    - The set of directed edges between partitions is defined as
      $$E_{P}=\left\{\left(P_{i},P_{j}\right)|\exists \left(v_{x},v_{y}\right)\in E,\;\mathrm{where}\;v_{x}\in P_{i},v_{y}\in P_{j},i\neq j\right\}$$
    - Each partition $P_{i}$ has an entering node $en(P_{i})$, defined as
      $$en\left(P_{i}\right)=\left\{v|v\in P_{i},\exists \left(v_{j},v\right)\in E,\;\mathrm{where}\;v_{j}\notin P_{i}\right\}$$
      This represents nodes in $P_{i}$ that receive input (tensor) values from nodes in other partitions.
    - Each partition $P_{i}$ has an exiting node $ex(P_{i})$, defined as
      $$ex\left(P_{i}\right)=\left\{v|v\in P_{i},\exists \left(v,v_{j}\right)\in E,\;\mathrm{where}\;v_{j}\notin P_{i}\right\}$$
      This represents nodes in $P_{i}$ that send output (tensor) values to nodes in other partitions.
- The deep learning low-level dispatcher $D_{LL}$ is defined as
  $$D_{LL}:\left(P,R,C\right)\to \left(P,R,C\right)$$
  where
  - A set of submodels or partitions of a DNN.
    $$P=\{P_{1},P_{2},\ldots ,P_{k}\}$$
  - A set of processing units (PUs) within a computer.
    $$R=\{R_{1},R_{2},\ldots ,R_{m}\}$$
  - A set of computational contexts associated with each partition $P_{i}$.
    $$C=\{C_{1},C_{2},\ldots ,C_{k}\}$$
  The low-level dispatcher performs the following actions.
  **Context Loading**  $\mathrm{LoadContext}(P_{i},R_{j},C_{k})$: Prepare the PU $R_{j}$ with the context $C_{k}$ for submodel $P_{i}$, including receiving input (tensor) values from other partitions before an entering node $en(P_{i})$ of partition $P_{i}$
  **Partition Execution**  $\mathrm{ExecuteModel}(P_{i},R_{j},C_{k})$: Initialize and manage the PU $R_{j}$ with the context $C_{k}$ for submodel $P_{i}$ between an entering node and an exiting node.
  **Context Saving**  $\mathrm{SaveContext}(p_{i},r_{j},c_{k})$: Save the PU $r_{j}$ with the context $c_{k}$ for submodel $p_{i}$, including sending input (tensor) values to other partitions after an exiting node $ex(P_{i})$ of partition $P_{i}$.

### 2.2. APIs for Deep Learning Dispatcher

A deep learning dispatcher can be implemented as a library, providing its functionality through API calls. These APIs enable the flexible management of DNN models and their partitions, tailored to the requirements of edge computing systems. A set of APIs for the deep learning dispatcher is as follows.

- load_neural_network and unload_neural_ network: These high-level dispatcher APIs manage the loading and unloading of a neural network.
- load_partition and unload_partition: These low-level dispatcher APIs handle the loading and unloading of neural network partitions on specific PUs.
- partition_create: This API is responsible for registering functions associated with the execution of the neural network partition.
- partition_input_handler and partition_output_handler: These APIs facilitate context switching such that context saving and context loading are achieved for the low-level dispatcher. partition_input_handler receives values from other partitions. It is implemented by reading values from memory or receiving values from networks. partition_output_handler sends values to other partitions. It is implemented by storing values in memory or sending values to networks.

### 2.3. Comparison Between Deep Learning Dispatcher and GPOS Dispatcher

Deep learning schedulers share both similarities and differences with general-purpose operating system (GPOS) schedulers. While both are responsible for efficiently managing resources such as processing units (PUs) to switch between tasks, deep learning schedulers focus specifically on switching between DNN models to optimize computationally intensive deep learning workloads. The deep learning dispatcher ($D_{HL},D_{LL}$) operates on top of the GPOS dispatcher, extending its functionality with DNN-specific optimizations.

The deep learning dispatcher is designed to adapt seamlessly to various edge computing architectures that incorporate multiple PUs. The high-level dispatcher ($D_{HL}$) and low-level dispatcher ($D_{LL}$) collaborate closely with the GPOS dispatcher, enabling their functionality to be executed across different PUs. This layered architecture ensures the efficient execution of DNNs over the GPOS dispatcher.

## 3. Case Studies

### 3.1. Application 1: An Example of Hierarchical Scheduler: PartitionTuner

This research primarily focuses on the dispatcher rather than the scheduling policy. However, to process deep neural networks efficiently, it is essential to integrate the dispatcher with an appropriate scheduling policy, as illustrated in Figure 2. We adopt the concepts of PartitionTuner, an operator scheduler for the deep learning compiler [13] to design both high-level and low-level scheduling policies for improving resource utilization and minimizing latency.

- A high-level scheduling policy
  A high-level scheduling policy provides the criteria for constructing a graph of submodels from the original DNN model. These criteria are generated by profiling functions in PartitionTuner along with other constraints such as resource availability, latency requirements, and task dependencies. When multiple DNN inference requests are made, the high-level dispatcher generates a graph of submodels according to the specified scheduling policy. If a specific processing unit (PU) is explicitly designated, the PU information is attached to the partition for execution by the low-level dispatcher. If no specific PU is designated, the PU information is included as a hint, allowing the low-level scheduling policy to make dynamic decisions regarding task execution. This policy ensures that DNN models are efficiently decomposed and prepared for execution in a scalable manner.
- A low-level scheduling policy
  A low-level scheduling policy dynamically assigns PUs within a computing system to optimize execution order and maximize resource utilization. It adjusts scheduling to allocate the required PUs during DNN partition execution, ensuring an optimal execution environment by constraining. This policy enhances PU utilization, reduces execution latency, and improves overall DNN performance. The effectiveness of both high-level and low-level scheduling techniques is demonstrated through case studies, showcasing their applicability in diverse, heterogeneous environments.
- Benchmarks
  We conducted experiments on the same environment used during the development of NEST-C, an open-source deep learning compiler [14], built upon the GLOW compiler framework [15]. The benchmark system consists of a heterogeneous computing platform featuring an ARM Cortex-A53 processor and a VTA (Versatile Tensor Accelerator) serving as the NPU [16].
  For evaluation, we measured the inference time of models trained on the ImageNet dataset. The evaluation was performed using seven pretrained CNN models, including ZFNet [17], AlexNet [18], GoogleNet [19], ResNet18/50 [20], ResNeXt [21], and SqueezeNet [22].
  Table 1 presents the inference times of these benchmark models, measured over 100 executions with different input images. The first and second columns list the model names and sizes, while the remaining columns compare inference times for execution on a CPU alone versus a CPU with an NPU. The last column shows the performance improvement ratio, calculated as CPU+NPU/CPU. On average, the application of a deep learning scheduler with an NPU improves performance by 51.6%.

### 3.2. Applications 2: Homogeneous Multiple NPUs

An illustrative system comprising multiple neural networks operating on multiple neural processing units (NPUs) was developed to evaluate the scalability and efficiency of the deep learning dispatcher. This system includes the construction of a hardware platform, schedulers with dispatchers, and application software.

**Figure 5:**
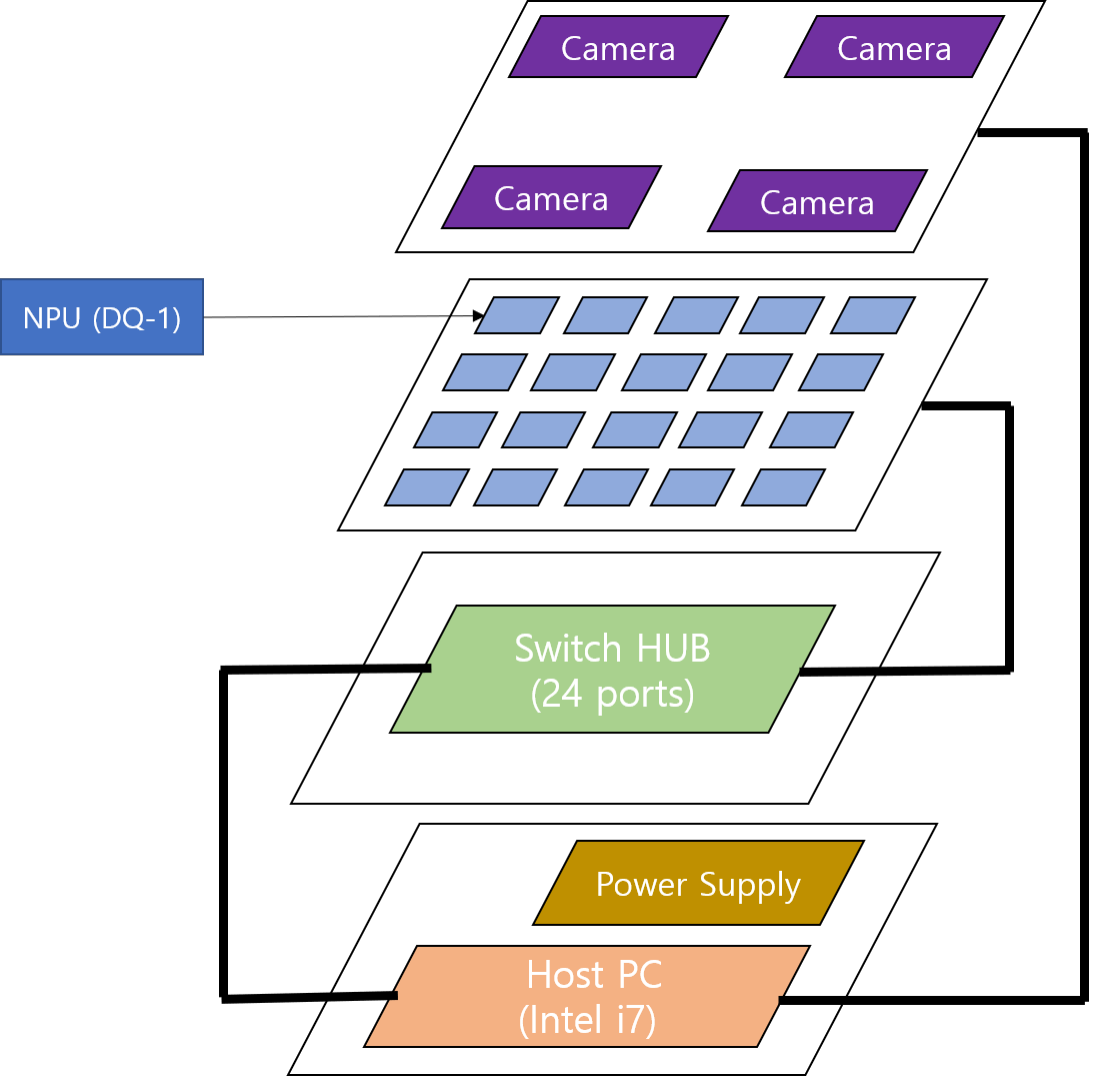
The overview of a hardware platform for multiple NPUs.

The hardware platform comprises 20 NPUs specifically designed for edge AI applications. Each NPU is integrated within a DQ-1 NPU SoC, co-developed by AiMFuture and LG Electronics, and includes a neural network accelerator, a CPU for general-purpose processing, and a DSP (Digital Signal Processor) for camera- and vision-related tasks. The 20 NPU boards are centrally coordinated by a Host CPU running Ubuntu Linux, which manages the entire system’s operations. The boards are organized in a 4x5-chip configuration and connected via Ethernet for communication, as illustrated in Figure 5.

**Figure 6:**
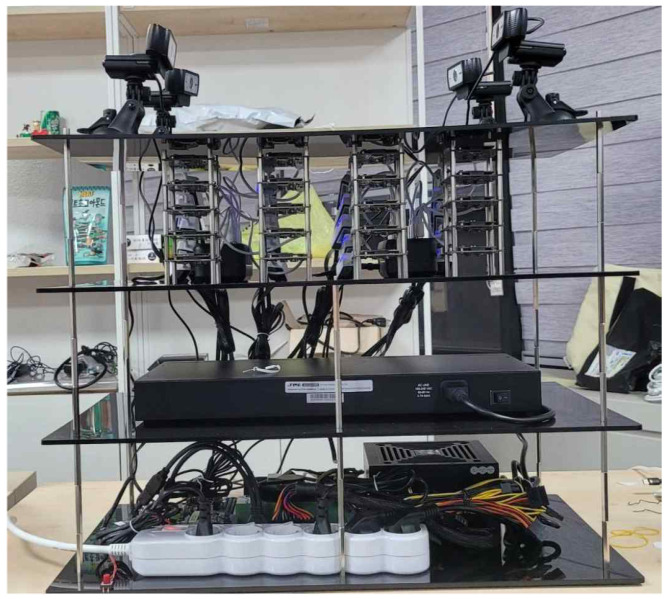
The hardware platform for multiple NPUs.

The partition is deployed on DQ-1 NPU SoC. The 20 DQ-1 boards are distributed on the third level of the hardware, connected through a USB-C-based power supply and Ethernet in Figure 6. A switch hub facilitates socket-based network communication between the DQ-1 boards and the Host CPU. Additionally, four camera modules positioned on the top level of the system are linked to a Host PC situated at the bottom level, enabling real-time image acquisition.

We evaluated the performance of seven artificial neural network applications (MNIST, LeNet-MNIST, ResNet18, ResNet50, MobileNet, SqueezeNet, and Inception) executing concurrently on multiple NPUs. Each application receives images for inference either by capturing them in real time from a connected camera or by reading them from storage devices.

**Figure 7:**
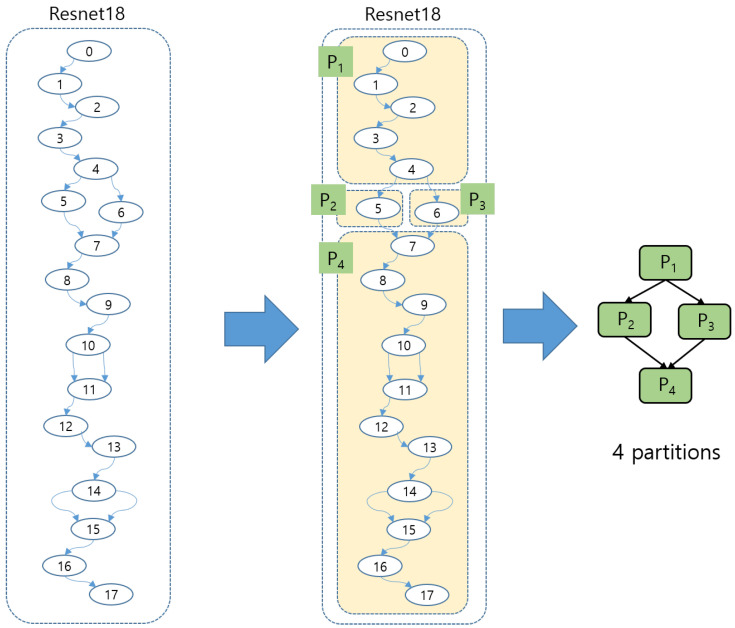
A partition graph derived from ResNet18.

Figure 7 illustrates an example of partitions from ResNet18.

**Figure 8:**
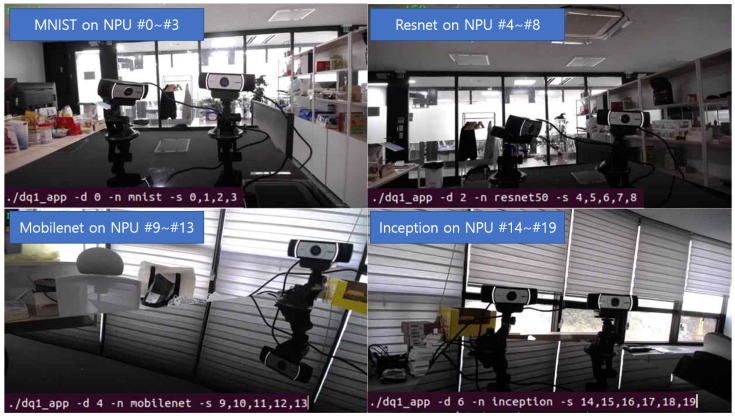
An example of inferring multiple DNNs on multiple NPUs.

Figure 8 illustrates the allocation of DQ-1 NPUs. Specifically, four NPUs (NPU #0-#3) are allocated for MINST, five NPUs (NPU #4–#8) for Resnet50, five NPUs (NPU #9–#13) for Mobilenet, and six NPUs (NPU #14–#19) for Inception. Each DNN application is connected to a distinct camera module, enabling concurrent inference. This allocation highlights the capability of the deep learning dispatcher to efficiently distribute and manage multiple DNN workloads across the 20 NPU boards.

**Figure 9:**
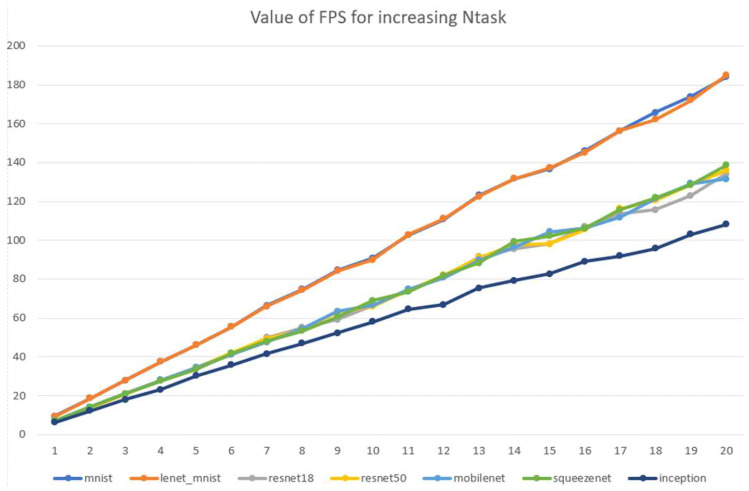
An increase in FPS by NPU boards utilized.

Figure 9 illustrates the scalability of the system, showing an increase in frames per second (fps) for each DNN as the number of NPU boards increases. A proportional improvement in fps was observed with the addition of more NPUs, though the rate of improvement varied by application. Computationally intensive neural networks, such as LeNet, MNIST and MobileNet, exhibited significant performance gains through parallelization. We believe that the integrated platform proposed in this study effectively distributes and processes large neural networks.

### 3.3. Application 3: Heterogeneous Multiple NPUs

**Figure 10:**
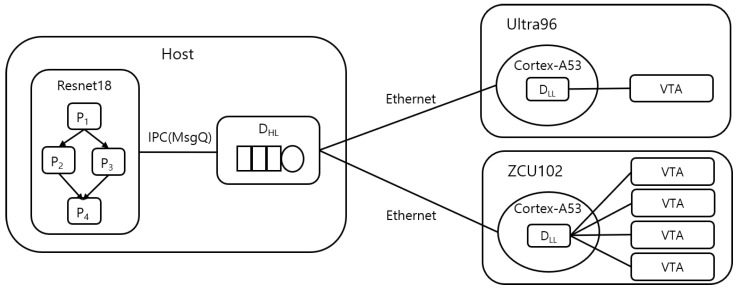
A hardware with heterogeneous multiple NPUs.

The second case study was conducted on a heterogeneous system composed of multiple NPUs based on Xilinx FPGAs, specifically the Ultra96 and ZCU102. The Ultra96 features an ARM Cortex-A53 processor and a single VTA as NPU, while the ZCU102, a more powerful FPGA, is equipped with an ARM Cortex-A53 processor and four VTAs. Figure 10 illustrates the hardware configuration of this setup.

The host system manages the high-level dispatcher, whereas NPUs, such as the Ultra96 and ZCU102, handle the low-level dispatchers. The placement of low-level dispatchers can vary. For example, both the high-level dispatcher and low-level dispatchers may be executed within the same or different computers.

This separation of dispatcher roles simplifies the implementation and ensures that the system can efficiently manage the distribution and execution of partitions across heterogeneous PUs. This design approach effectively leverages the unique capabilities of each PU, optimizing performance and scalability.

Deep learning scheduling policies can vary depending on the scheduling objectives. To illustrate this, we will present an example where the scheduling policy is designed to achieve the objective of minimizing latency time. This example demonstrates how a specific scheduling policy can be combined with dispatchers to optimize performance by reducing the latency time.

Resnet18 in Figure 7 has four partitions. The latency time is composed of partition computation time and context switching time.

$$\mathrm{latency}\;\mathrm{time}=max(\sum \left(T_{compuation}+T_{context\_switch}\right))$$

where

- $T_{computation}$ is the computation time for a partition node.
- $T_{contextswitch}$ is the communication time between nodes, including the context saving time and context loading time.

**Figure 11:**
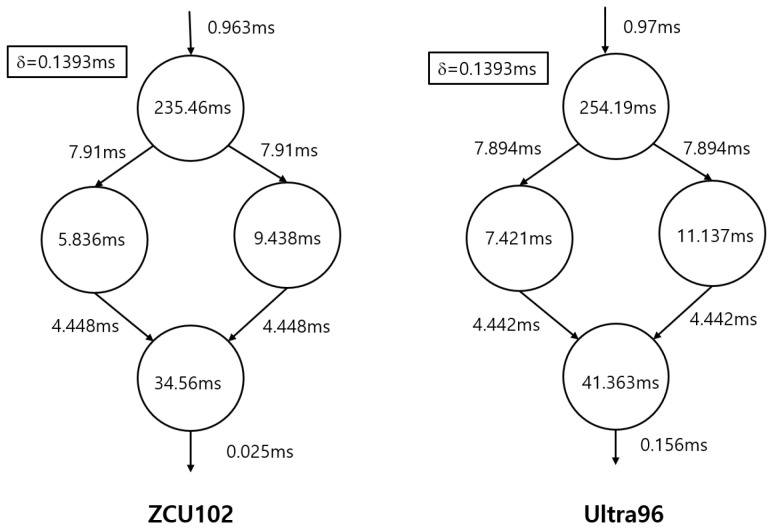
Computation time and context switching time for Resnet18.

Figure 11 illustrates the partition graph for the ZCU102 and Ultra96 devices, incorporating both computation and context switching times. The parameter δ, representing the latency time in a device, is displayed in the upper-left corner of each graph. Using this partition graph, the inference latency of the DNN can be readily calculated.

**Figure 12:**
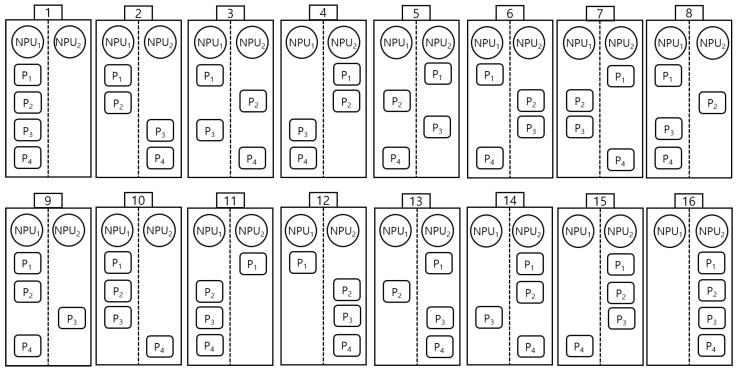
The sixteen possible schedules for four partitions on two FPGAs.

For ResNet18, the model is divided into four partitions for execution. The system provides 16 possible schedules for mapping these four partitions across the two FPGAs, as shown in Figure 12. The inference latency for each schedule is calculated using a latency analytic model. Among these schedules, schedules #1, #8, and #9 achieve the smallest latency times. Consequently, one of these three schedules could be selected to optimize inference performance based on the scheduling objective.

In this demonstration, a simple analytic model was used for calculating latency. However, if a more precise analytic model is developed, it could be easily integrated with the dispatcher, further enhancing the system’s performance.

## 4. Conclusions and Future Research

This paper presented a hierarchical dispatcher architecture for efficiently managing the execution of multiple DNNs on edge devices equipped with heterogeneous PUs. The proposed architecture demonstrates both scalability and flexibility by incorporating high-level and low-level dispatchers. The high-level dispatcher is responsible for constructing partition graphs of submodels, enabling the efficient decomposition and global scheduling of complex DNNs. In parallel, the low-level dispatcher allocates submodels to specific PUs, ensuring optimal resource utilization and seamless integration with various scheduling policies.

Through case studies on both homogeneous and heterogeneous NPU configurations, the dispatcher architecture was shown to be effective in distributing DNN partitions across multiple edge devices, supporting the concurrent execution of multiple models. This research emphasizes the importance of decoupling dispatcher responsibilities into modular high-level and low-level components, a design that simplifies implementation and enhances portability across diverse hardware environments.

For future work, we plan to investigate the integration of more advanced scheduling policies, particularly those that consider power efficiency and real-time constraints in large-scale DNN deployments on commercial platforms such as Intel Core Ultra and Qualcomm Snapdragon. Moreover, we aim to reduce communication overhead by optimizing partition placement by allocating consecutive partitions to the same PU, such as cross-layer optimization. This approach would eliminate unnecessary data transfers at partition boundaries, thus improving overall execution efficiency and lowering latency in distributed edge computing environments.

Another direction for future research is to improve the generality of the dispatcher by applying it to a broader range of model types, including large language models (LLMs). While the dispatcher is designed to be model-agnostic and theoretically applicable to LLMs, further work is required to implement and validate its performance across diverse deep neural network architectures. By applying the dispatcher to various types of DNNs, we aim to demonstrate its adaptability and effectiveness beyond vision-centric models, ultimately extending its utility in large-scale and heterogeneous AI computing environments.

We believe this research can serve as a foundational framework for the development and integration of DNN scheduling policies, particularly in heterogeneous and resource-constrained computing environments.

## Figures and Tables

**Table 1:** A benchmark for Application 1: Scheduling Policy.

Benchmarks		Time (μs)		Performance
**Name**	**Size (Mbytes)**	**Baseline (CPU)**	**CPU + NPU**	**Improvement (%)**
ZFNet	349	2435.17	1694.38	69.6
AlexNet	244	860.10	810.73	94.3
GoogleNet	171	1465.80	482.30	32.9
ResNet50	103	3183.74	636.19	20.0
ResNeXt50	100	6638.85	3959.94	59.6
ResNet18	47	1269.18	249.03	19.6
SqueezeNet	5	298.64	195.46	65.5

## Data Availability

The original data presented in the study are openly available in https://github.com/etri/nest-os (accessed on 30 March 2025).
